# Replacing Di(2-ethylhexyl) Terephthalate by Di(2-ethylhexyl) 2,5-Furandicarboxylate for PVC Plasticization: Synthesis, Materials Preparation and Characterization

**DOI:** 10.3390/ma12142336

**Published:** 2019-07-23

**Authors:** Marina Matos, Rosemeyre A. Cordeiro, Henrique Faneca, Jorge F. J. Coelho, Armando J. D. Silvestre, Andreia F. Sousa

**Affiliations:** 1 CICECO-Aveiro Institute of Materials and Department of Chemistry, University of Aveiro, 3810-193 Aveiro, Portugal; 2 Center for Neuroscience and Cell Biology, University of Coimbra, 3004-504 Coimbra, Portugal; 3 CEMMPRE, Department of Chemical Engineering, University of Coimbra, 3030-790 Coimbra, Portugal

**Keywords:** polymers, PVC, sustainable plasticizers, non-toxic, 2,5-furandicarboxylic acid

## Abstract

The worldwide regulatory demand for the elimination of non-phthalate compounds for poly(vinyl chloride) (PVC) plasticization has intensified the search for alternatives. Concomitantly, sustainability concerns have highlighted sugar-based 2,5-furandicarboxylic acid as one key renewable-chemical for the development of several products, namely di(2-ethylhexyl) 2,5-furandicarboxylate (DEHF) plasticizer. This study addresses the use of DEHF under a realistic scenario of the co-existence of both DEHF and entirely fossil-based plasticizers. More precisely, original PVC blends using mixtures of non-toxic DEHF and di(2-ethylhexyl) terephthalate ester (DEHT) were designed. The detailed structural, thermal, and mechanical characterization of these materials showed that they all have a set of interesting properties that are compatible with those of commercial DEHT, namely a low glass transition (19.2–23.8 °C) and enhanced elongation at break (up to 330%). Importantly, migration tests under different daily situations, such as for example exudation from food/beverages packages and medical blood bags, reveal very low weight loss percentages. For example, in both distilled water and phosphate buffered saline (PBS) solution, weight loss does not exceed ca. 0.3% and 0.2%, respectively. Viability tests show, for the first time, that up to 500 μM of DEHF, a promising cytotoxic profile is observed, as well as for DEHT. Overall, this study demonstrates that the combination of DEHF and DEHT plasticizers result in a noticeable plasticized PVC with an increased green content with promising cytotoxic results.

## 1. Introduction

Poly(vinyl chloride) (PVC) is a thermoplastic polymer known since the XIX century, but today it is still one of the most widely used thermoplastic polymers in respect to worldwide plastic consumption. In fact, according to a recent market study [1], in 2016, over 42 million tons of PVC were consumed, corresponding to over 16% of total plastics demand, and it is continuously growing. PVC is routinely plasticized in order to increase its flexibility or workability [2,3], and thus decreases the melting (*T_m_*) and glass transition (*T_g_*) temperatures, as well as the elastic modulus in order to meet the requirements of several applications in food/beverage packaging, dialysis bags, blood bags, tubing systems, and children toys, among many others [4]. Free plasticizers are relatively low molecular weight molecules that interact with PVC electrophilic C-Cl groups, mainly in the amorphous regions, reducing polymer-polymer chains interactions. For decades the most used plasticizers were esters of phthalic acid derivatives [5], mainly di(2-ethyl-1-hexyl) orthophthalate ester (DEHP or DOP). However, its use has raised several concerns associated with migration issues overtime and especially due to the severe adverse health effects (especially when it enters the blood stream) [6]. In fact, DEHP has been banned in toys and childcare articles, and its broader use in articles has been restricted to a low concentration in the European Union (EU) [7] and in several other countries. Consequently, non-phthalate plasticizers have become more widely used (see Reference [8] and references therein). This is the case for di(2-ethylhexyl) terephthalate ester (DEHT), DEHP structural isomer, but it is not associated with toxic effects [6]. However, the DEHT plasticizer (or even DEHP) is derived from fossil resources.

In recent years, motivated by increasing attention to sustainability awareness and concerns regarding the massive use of petroleum-based products [9] (including plasticizers) has called for a paradigm shift towards the development of renewable-based ones, or at least partially renewable ones. In this vein, several alternatives to DEHT and toxic DEHP have been disclosed [10,11], namely tung oil- [12,13], cardanol- [14,15], poly(caprolactone)- [10], or poly(hexane succinate)-based [11] plasticizers. 2,5-Furandicarboxylic acid (FDCA) is a well-recognized sugar-based monomer that is structurally related with terephthalic acid (TPA) and the precursor of several polyesters with thermal and mechanical properties very similar to those prepared from fossil-TPA [9,16]. Moreover, unlike phthalates, furan compounds are easily metabolized and FDCA itself is a common human urinary metabolite [17]. Therefore, its use in broader applications, including as a plasticizer, besides in polymer synthesis, is of upmost interest. However, there are only a few reports on the synthesis of 2,5-furandicarboxylate esters plasticizers and its use in PVC blends [18,19], specifically on the synthesis of di(2-ethylhexyl) 2,5-furandicarboxylate (DEHF) to mimic the commercial benchmark-DEHT [18,19]. Despite the fact that 2-ethyl-1-hexanol is mainly produced from fossil resources, its synthesis can also be based on renewable resources starting from, for example, *n*-butanol (which can be renewable-based) [20], thus enabling an entirely renewable-based DEHF.

Previous studies on PVC-DEHF blends (between 10–50 phr of plasticizer) were shown to have promising elongation at the break within 57%–249% (slightly lower than DEHP), a *T_g_* between 1–59 °C; to be thermally stable up to 188–225 °C; and the migration in hexane was at most around 9% [19]. Despite these relatively promising mechanical and thermal properties, some important ones like DEHF cytotoxicity, migration studies using broader spectra of model solvents (including water), and the volatile resistance behavior remain unknown, as well as biodegradation studies. Moreover, in a more realistic scenario, at the industrial scale, the replacement of petroleum-based DEHT with higher contents of renewable counterparts will be hampered due to relevant economic issues related to cost-competitiveness. In this vein, the partial replacement of DEHT by DEHF is a logical approach worth exploring in order to increase the designated “green-content” [21].

Therefore, in this study, different ratios of DEHF/DEHT were used to assess the partial replacement of fossil-based DEHT by more sustainable DEHF in PVC formulations (up to 36 wt %, 20 phr), and increase the compatibility/affinity of the mixture of plasticizers with the PVC matrix. These new PVC blends were characterized in detail using ATR FTIR to probe PVC-plasticizer compatibility; XRD assessed the amorphous/crystallinity nature of the blends and together with TGA, DMTA and tensile tests were used to assess their thermal stability and mechanical properties to confirm PVC-plasticizer compatibility. The migration stability of the plasticizers was studied through leaching and volatile resistance tests to further evaluate their use under relevant practical conditions. Additionally, in this study, for the first time, the cytotoxicity of DEHF was evaluated in terms of cell viability tests in order to foresee its wide application as a benign PVC plasticizer.

## 2. Materials and Methods

### 2.1. Materials

2,5-Furandicarboxylic acid (>98%) was purchased from TCI Europe NV (Zwijndrecht, Belgium). 2-Ethyl-1-hexanol (≥99.6%), stearic acid (95%), anhydrous sodium sulfate (≥99.0%), zinc stearate purum, and Dulbecco’s modified Eagle’s medium—high glucose (DMEM-HG) were supplied by Sigma Aldrich-chemicals Corp. (Sintra, Portugal). Sulfuric acid (96%), sodium chloride (≥99.0%), and deuterated chloroform (99.8% D) were acquired from Acros Organics (Geel, Belgium). Chloroform and cyclohexane (HPLC grade) were purchased from Fisher Scientific (Porto Salvo, Portugal and Panreac Applichem (Barcelona, Spain), respectively. Activated carbon (granules with density of 2 g cm^−3^) was obtained from VWR Chemicals. Di(2-ethyl-1-hexyl) 1,4-terephthalate (DEHT) (DOTP-168 Eastman) was supplied by Eastman Chemical Company (Madrid, Spain) and PVC resin (VICIR S1200; The K-Fikentscher value of 70, corresponding to number-average molecular weight equal to 59000 and a polydispersity of 1.97) [22], was provided from CIRES, Lda. (Portugal). All chemicals were used as received, without further purification.

### 2.2. Synthesis of Di(2-ethyl-1-hexyl) 2,5-Furandicarboxylate (DEHF)

In this study, DEHF was synthesized via Fisher esterification following an adapted procedure described elsewhere [23,24]. Briefly, 5.0 g of FDCA (32.0 mmol) and 25.0 g of 2-ethyl-1-hexanol (192.2 mmol) (diacid:diol, 1:6 mol/mol) were reacted in the presence of concentrated sulfuric acid (1 wt %, total diacid weight) and was kept at 160 °C for 6 h. The resulting reaction product was washed with an aqueous NaCl solution (30% m/v) until it reached pH 7, and was then extracted with chloroform. The extracted DEHF was dried and weighed. DEHF was isolated as a light-yellow liquid at room temperature in a 94% yield. The purity and molecular structure of the isolated compound was also confirmed by ATR FTIR (Figure S1), NMR (Figures S2 and S3), and GC-MS analyses (Figure S4), and was in accordance with previously reported data [19]. ATR FTIR (ν/cm^−1^): 3126 (=C-H); 2957, 2927, and 2859 (υ C-H, methylene and methyl groups); 1719 (C=O); 1581 (C=C); 1461, 1380, 1271, and 1220 (C-O); 1017 (furan ring breathing); 968, 822, and 764 (2,5-dibustituted furan ring) (Figure S1). ^1^H NMR (300 MHz, CDCl_3_, *δ*, ppm): 7.20 (s, 2H, H3, H4), 4.25–4.28 (2d, 4H, H6), 1.69–1.77 (m, 2H, H7), 1.30–1.50 (m, 16H, H10, H11, H12), 0.92–0.98 (2t, 12H, H9, H13) (Figure S2). ^13^C NMR (75 MHz, CDCl_3_, *δ*, ppm): 158.3 (2,5- C=O); 147.0 (C2/C5); 118.1 (C3/C4); 67.9 (C6); 38.8 (C7); 30.3 (C10); 28.9 (C11); 23.8 (C8); 22.9 (C12); 14.0 (C13); 11.0 (C9) (Figure S3). MS (EI) m/z (relative intensity %): 380 ${\left[M\right]}_{.}^{+}$ (1), 269 (17), 251 (14), 223 (5), 157 (100), 112 (31), 70 (35), 57 (9) (Figure S4).

### 2.3. Preparation of PVCs

Plasticized PVC films were prepared by pre-mixing PVC-K70 resin (100 per hundred resin (phr)), stearic acid (0.3 phr), zinc stearate (1 phr), and a mixture of DEHT/DEHF plasticizers in different relative amounts (Table 1) for ca. 5 min and vigorous hand-stirring. Subsequently, the ensuing mixtures were allowed to rest for 30 min, and after that period, they were mixed using a two-roll mill (Collin machine type W-150P, Collin, Ebersberg, Germany), at 140 °C and 1600 rpm for 5 min. The mixtures were compression-molded (Carver press Model 3851Carver Inc., Wabash, IN, USA) using a steel mold (110 mm × 110 mm × 2 mm) and heated until reaching 140 °C, and after standing for 2 min, pressed at 28 tons until a thickness of 2 mm was reached, and finally depressed and fast cooled to room temperature.

### 2.4. Characterization

The viscosity and density of DEHF plasticizer were measured using an automated SVM 3000 Anton Paar rotational Stabinger viscometer-densimeter (Anton Paar, Graz, Austria) at atmospheric pressure, within the temperature range of 293.15 to 323.15 ± 0.02 K.

Attenuated total reflectance Fourier transform infrared (ATR FTIR) spectra were obtained using a PARAGON 1000 Perkin-Elmer FTIR spectrometer (Perkin-Elmer, Waltham, MA, USA) equipped with a single-horizontal Golden Gate ATR cell. The spectra were recorded after 128 scans, at a resolution of 4 cm^−1^, within the range of 500 to 4000 cm^−1^. The ATR FTIR spectra of all samples were normalized relative to the vibrational peak at 2953 cm^−1^.

^1^H and ^13^C NMR spectra were recorded using a Bruker AMX 300 spectrometer (Bruker, Billerica, MA, USA), operating at 300 or 75 MHz, respectively. All chemical shifts (*δ*) were expressed as parts per million, downfield from tetramethylsilane (used as the internal standard).

Gas chromatography-mass spectrometry (GC-MS) analyses were performed using a Trace gas chromatograph (2000 series, Thermo Fisher Scientific, Waltham, MA, USA) equipped with a Thermo Scientific DSQ II mass spectrometer (Waltham, MA, USA). Separation of compounds was carried out in a DB-1 J&W capillary column (30 m × 0.32 mm inner diameter, 0.25 μm film thickness, Thermo Fisher Scientific, Waltham, MA, USA) using helium as the carrier gas (35 cm s^−1^). The chromatographic conditions were as follows: Initial temperature equal to 80 °C for 5 min, then the temperature was raised up to 260 °C, at 4 °C min^−1^ rate, and finally, up to 285 °C, at 2 °C min^−1^ and maintained for 8 min. The injector temperature was 250 °C transfer-line temperature equal to 290 °C, and split ratio of 1:33. The mass spectrometer was operated in the electron impact (EI) mode with an energy of 70 eV and data were collected at a rate of 1 scan s^−1^ over a range of m/z 33–700. The ion source was kept at 250 °C. The sample was prepared dissolving DEHF in chloroform (1 mg mL^−1^).

X-ray diffraction (XRD) analyses were performed using a Philips X’pert MPD diffractometer (Malvern Panalytical, Malvern, UK) operating with CuKα radiation (λ = 1.5405980 Å) at 40 kV and 50 mA. Samples were scanned in the 2*θ* range of 5 to 50°, with a step size of 0.04°, and time per step of 50 s.

Thermogravimetric analysis (TGA) were carried out with a Setaram SETSYS analyzer (Setaram, Caluire, France) equipped with an alumina plate. Thermograms were recorded under a nitrogen flow of 20 mL min^−1^ and heated at a constant rate of 10 °C min^−1^ from room temperature up to 800 °C.

Dynamic mechanical thermal analyses (DMTA) were performed with a Tritec 2000 DMA Triton (Triton Technology Ltd., Mansfield, MA, USA), operating in tension mode, except for pure PVC, for which a material pocket accessory was used, operating in the single cantilever mode. Tests were performed at 1 and 10 Hz and the temperature was varied from −100 to 150 °C, at 2 °C min^−1^. The glass transition temperature (*T_g_*) was determined as the maximum peak value of tan *δ*.

Tensile tests were obtained with an Instron 5564 tensile testing machine (Instron, Norwood, MA, USA) at a cross-head speed of 10 mm min^−1^ using a 500 N static load cell. The tensile test specimens were rectangular strips (50 mm × 10 mm × 2 mm) pre-conditioned for 72 h, at 25 °C. Each measurement was repeated at least five times.

### 2.5. Migration Resistance Assays

The leaching tests were performed in agreement with the Standard Test Method ASTM D 1239-98 [25]. PVC-DEHF/DEHT specimens (10 mm × 10 mm × 2 mm) were pre-conditioned at 23 ± 2 °C with a humidity of 50 ± 5% for 24 h. After this period, the specimens were immerged in 10 mL of the chosen solvent (distilled water, sodium phosphate buffer at pH ~7, or cyclohexane) at 23 ± 2 °C for 48 h. The films were then removed from the liquid, washed thoroughly with distilled water, and dried. Each measurement was repeated at least three times. The weight loss percentage was calculated using the expression: $Weight\;loss\;\left(\%\right)=\left(W_{i}-W_{f}\right)/W_{i}\times 100$ where *W_i_* and *W_f_* stand for the specimen weights prior and after leaching resistance tests, respectively.

Volatile resistance tests were performed according to the international standard ISO 176-2005 [26] to determine the loss of plasticizers through the activated carbon method. PVC-DEHF/DEHT specimens (10 mm × 10 mm × 2 mm) were immersed at the center of activated carbon at 40 °C, for 48 h. After this period, samples were washed with distillate water and dried. Each measurement was repeated at least three times. The weight loss was determined using the same equation represented above.

### 2.6. Cytotoxicity Assays

The cytotoxicity of the plasticizers was evaluated in the 3T3-L1 cell line acquired from ATCC. For this purpose, 35 × 103 3T3-L1 cells were seeded onto a 48-well culture plate 24 h prior to incubation with the compounds (cells were used at 70% confluence). Then, cells were incubated with different concentrations of the plasticizers for 48 and 72 h, and the cell viability was assessed by a modified Alamar Blue assay [27]. This assay measures the redox capacity of the cells due to the production of metabolites as a result of cell growth. Briefly, the cell culture medium of each well was replaced with 0.3 mL of DMEM-HG containing 10% (v/v) of Alamar Blue (0.1 mg mL^−1^ in phosphate buffered saline), and after 1 h of incubation at 37 °C, 170 μL of the supernatant were collected from each well and transferred to 96-well plates. The absorbance was measured at 570 and 600 nm in a SPECTRAmax PLUS 384 spectrophotometer (Molecular Devices, Union City, CA, USA). Cell viability was calculated as a percentage of the control cells (cells not treated with the plasticizers) according to the ratio: ${\left(A_{570}-A_{600}\right)}_{treated\;cells}/{\left(A_{570}-A_{600}\right)}_{control\;cells}\times 100$. The data are expressed as mean ± standard deviation obtained from n = 9 (from three independent experiments).

## 3. Results and Discussion

The partial replacement of fossil-based DEHT plasticizer in PVC formulations was accomplished by preparing several mixtures incorporating the more sustainable DEHF in increasing amounts. These plasticizers were chosen due to the promising properties of the DEHF-PVC blends [18,19], as well as because DEHT is a commercial plasticizer with low toxicity (compared to the phthalates homologues, e.g., DEHP) [6]. The DEHF/DEHT relative amounts were varied between 0/100 wt % reaching 64/36 wt % (Table 1).

**Scheme 1:**
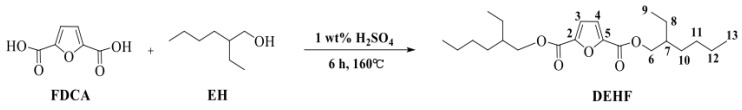
Synthesis of DEHF.

The preparation of the novel plasticized PVCs comprised, in the first step, DEHF synthesis by a Fisher esterification of FDCA and 2-ethyl-1-hexanol, under acidic conditions (Scheme 1), and its structure probe by ATR FTIR, NMR, and MS spectroscopies (Figures S1–S4 of Supplementary Materials) with a molecular weight equal to 380.30 (Table S1) and in high purity (ca. 100% from gas chromatography analysis). This was followed by, in the second step, a DEHF mixture with DEHT in different relative amounts (0–36 wt % of DEHF) (Table 1), and then, finally, compounding the plasticizers (in 55 wt %, in agreement with other work ranges [23,28,29]) with PVC resin, and also with stearic acid and zinc stearate, selected as a lubricant and heat stabilizer, respectively [30,31].

Interestingly, it was noted that DEHF has the same density (0.98) but slightly lower viscosity (55 cP) than DEHT (63 cP) (Table S1). DEHF/DEHT blends were completely miscible, in accordance with their good compatibility demonstrated ahead by ATR FTIR and further confirmed by their full characterization.

### 3.1. Structural Characterization of the PVCs

**Figure 1:**
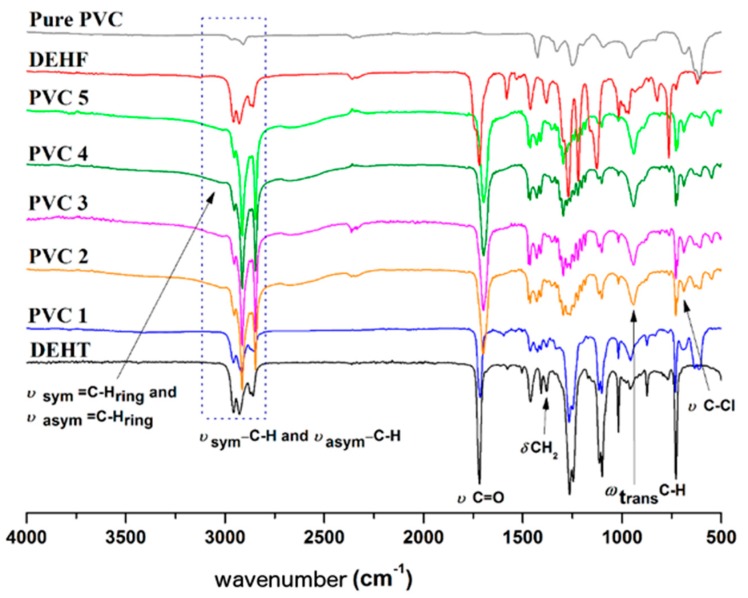
Normalized ATR FTIR spectra of all PVC films, and DEHF and DEHT plasticizers.

The PVC-DEHF/DEHT films and their main components, i.e., DEHF and DEHT plasticizers, as well as the pure PVC, were studied by ATR FTIR spectroscopy (Figure 1).

The spectrum of DEHF is in accordance with its expected structure, and with a previous report [19], displaying: symmetrical and asymmetrical C-H stretching vibrations of the furanic ring (ν*_sym_* =C-H_ring_ and ν*_asym_* =C-H_ring_) near 3141 and 3162 cm^−1^ (Figure S1); symmetrical and asymmetrical C-H stretching of the methyl and methylene groups of the EH moieties (ν*_sym_* -C-H and ν*_a__sym_* -C-H) at 2928, 2958, 2860, and 2873 cm^−1^, respectively [19,29,32]; one intense band was at 1719 with a shoulder at around 1740 cm^−1^ arising from the carbonyl stretching vibration, typical of ester groups (ν C=O).

The presence of the higher wavenumber band at 1740 cm^−1^ is most likely associated with plasticizer-plasticizer associations involving the carbonyl groups [33]. CH_3_ was also detected in plane deformation (δ CH_3_) at 1380 cm^−1^; aromatic C-H in plane angular deformation vibration at 1270 cm^−1^; near 941 cm^−1^ the out of plane trans deformation vibration (ω C-H) [19]; and the typical vibrational modes of 2,5-disubstituted furanic ring near 980, 823, and 764 cm^−1^. The spectrum of the fossil-based DEHT is quite similar to that of its DEHF counterpart, except for the bands at 3100 cm^−1^, arising from the C-H stretching mode of the benzenic ring and those of 1,4-disubstituted benzenic ring at 728 cm^−1^.

The spectrum of the pure PVC showed the main characteristic vibrational bands of this polymer (Figure 1), viz.: C-H stretching on neighboring C-Cl group at 2966 cm^−1^; asymmetrical and symmetrical C-H stretching (ν*_asym,sym_* -C-H) at 2908 cm^−1^; CH_2_ deformation at 1425 cm^−1^; CH_2_ deformation at 1328 cm^−1^, Cl-CH out of plane angular deformation at 1252 cm^−1^; out of plane trans deformation at 958 cm^−1^ and the C-Cl bond stretching vibrations in the region between 660–580 cm^−1^ [34,35,36].

As expected, the ATR FTIR spectra of the PVC-DEHF/DEHT blends (Figure 1) displayed the characteristic bands of the two main components, i.e., the plasticizers and the pure PVC. The characteristic vibrational peaks of the other additives were not distinguishable in the spectra of the films.

#### Probing PVC-Plasticizer Compatibility

It is well known that an enhanced compatibility/miscibility of a polymer-plasticizer blend is essential for the effective plasticization of the polymer and related thermal and mechanical properties, as well as for the migration resistance of these chemicals. Thus, probing PVC-plasticizer compatibility resulting from physical interactions between the components of the mixture was a fundamental step taken in this study using infrared spectroscopy [3,37].

**Figure 2:**
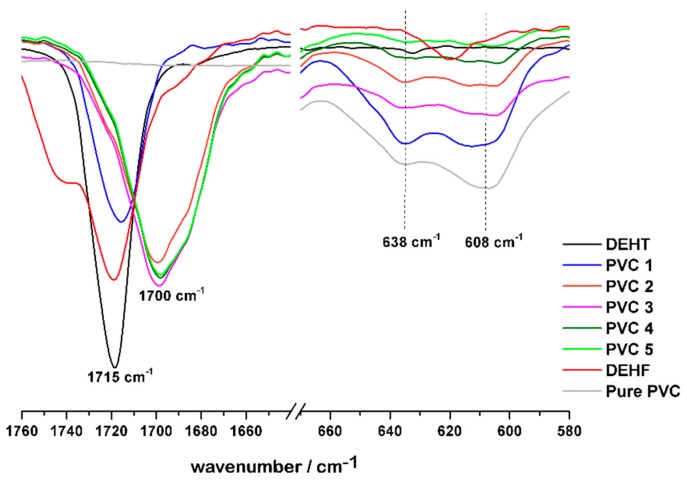
ATR FTIR spectra of all PVC-DEHF/DEHT blends in the C=O and C-Cl stretching regions.

As previously suggested, in the particular case of PVC-aromatic ester plasticized blends, for example, PVC-DEHT or PVC-DEHP, the main interactions are essentially of dipole-dipole nature involving the polarized carbon−chlorine (C-Cl) and the carbonyl (C=O) groups of PVC and plasticizer, respectively [29,33,36]. The vibrational modes stemming from these groups denounced the blends compatibility and will be studied in more detail. From the neat plasticizers to the ensuing blends, a red-shift of the C=O stretching band from 1718 cm^−1^ to lower wavenumbers, e.g., to near 1700 cm^−1^ for all the PVC2-5 blends and only to 1715 cm^−1^ for PVC1, was observed (Figure 2) in accordance with the findings of Tabb et al. [36]. The same was not observed in the case of the mixture composed exclusively of the plasticizers (Figure S1), showing instead the C=O stretching band centered near 1719 cm^−1^.

This result is indicative of interactions between the plasticizers and the PVC polymer chains and is in accordance with an effective plasticization process [29,32,38]. However, in a previous study by Yu et al. [19] about PVC-DEHF films, no shift of this band was observed, which was attributed to a probable weak interaction between the plasticizer and the PVC matrix. Oppositely, the results herein presented support the enhanced compatibility of the present PVC plasticization system involving both DEHF and DEHT. Both the nature of plasticizers mixture and the methodology used to prepare the PVC formulations, by a pre-mixture procedure between all the components for a period of 30 min, contribute to the enhanced compatibility observed.

Other important spectral changes, in the region between 660–580 cm^−1^ (Figure 2), attributed to the C-Cl stretching vibration modes (*ν* C-Cl), were clearly observed. There was both a broadening and a splitting of the band associated with the atactic PVC fraction (amorphous domains), centered at 608 cm^−1^, especially in the case of the blends prepared with higher amounts of DEHF compared with neat PVC. Furthermore, the band at 638 cm^−1^, related to crystalline domains of PVC, remained essentially unchanged, except for DEHF amounts higher than 27% (PVC4). Similar observations were denoted in several other previous studies, and were pointed out as evidence of a strong interaction between PVC and the plasticizer [14,29,39]. Moreover, this is consistent with the classical plasticization process essentially affecting the amorphous domains of PVC, through a solvation process [36]. Hence, for the present PVC-DEHF/DEHT system, similar conclusions can be inferred. In particular, it reveals that the DEHF/DEHT mixture of plasticizers could improve the plasticization process (even compared to the single use of DEHT in PVC1), thus increasing the compatibility between the different compounds in the PVC formulations.

**Figure 3:**
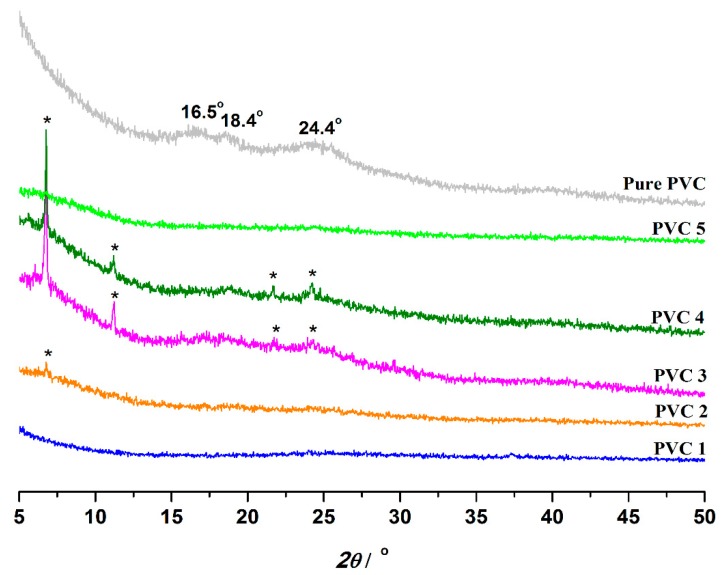
X-ray diffractograms of all DEHF/DEHT-based PVC films and pure PVC. * indicates the zinc stearate diffraction peaks.

The XRD patterns of all plasticized films (Figure 3) evidenced their essentially amorphous nature, with, nonetheless, some broad crystallinity peaks at 2*θ* ≈ 16.5, 18.4, and 24.4°, in agreement with the literature and ATR FTIR results [40,41]. The pure PVC pattern was consistent with the PVC-DEHF/DEHT ones, although evidencing a higher degree of crystallinity.

### 3.2. Mechanical Properties of the PVCs

**Figure 4:**
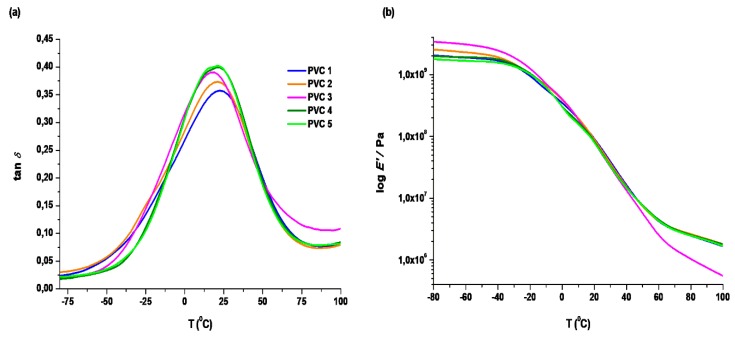
Main results of DMTA analyses (**a**) tan *δ* and (**b**) *E′* traces of all plasticized PVC films.

All blends were analyzed by DMTA to evaluate the influence of using DEHF/DEHT plasticizers on their dynamical-mechanical properties. Figure 4 shows the tan *δ* and storage modulus (*E′*) traces of DEHF/DEHT-based PVC films, recorded in tension mode, at constant frequency (1 Hz). Figure S5 of Supplementary Materials displays the loss modulus (*E″*) traces. The main results are summarized in Table S2.

The tan *δ* curves (Figure 4a) of all plasticized PVC films displayed a single maximum corresponding to a *α* transition, ascribed to the glass transition (*T_g_*), with values ranging from 20.4 to 23.5 °C, typically slightly decreasing with the increasing content of DEHF in the blend (PVC1-3). However, going into further detail, in the case of those blends incorporating the highest relative amounts of DEHF (PVC4 and 5), the *T_g_* remains roughly constant. This could be related to the existence of more rigid DEHF molecules in the blends that play, at the upper limits, an effective plasticization role, as described before [19], leading to no further decrease of the *T_g_* values. Indeed, the intrinsic hardness of the furan ring brings rigidity to the products thereof, such as, for example, in the case of FDCA-based polyesters [42,43]. A similar effect was also noted in mechanical properties, as discussed below. Therefore, PVC3 presented the optimum plasticization results.

In comparison with the tan *δ* curve of pure PVC, the maximum was at much higher temperatures than in the case of the blends, approximately 97.4 °C. These results are in accordance with literature results on pure PVC and on other plasticized systems incorporating DEHF or other 2,5-FDCA-based esters [18,19], DEHT [13,44], and binary mixtures of DEHT with other plasticizers [32,45]. Importantly, the appearance of a single *T_g_* on the tan *δ* traces of plasticized PVC films, and shifted to lower values, reaching a difference of ca. 76 °C for PVC2-5 and slightly less to ca. 74 °C for PVC1, suggested compatibility between the plasticizers and PVC, as ATR FTIR results already indicated.

The blends’ storage modulus traces (Figure 4b) clearly show three main regions, viz.: One region below the glass transition corresponding to an almost constant high modulus, ranging from 551–695 MPa at −10 °C; followed by an abrupt decrease of the modulus due to *T_g_* transition; finally, at higher temperatures, the modulus reached its minimum and was maintained roughly constant between 38 to 43 MPa at 25 °C.

A general improvement of the flexibility of the PVCs was noted, accordingly having a storage modulus far below that of pure PVC (e.g., 12–13 MPa at 40 °C vs. 377 MPa at 40 °C, respectively) [46]. However, comparing the use DEHF/DEHT mixtures of plasticizers (PVC2-5) with the single use of DEHT (PVC1), *E′* and *E″* were higher in the former case, most probably due to the incorporation of the stiff furan plasticizer (Table S2 and Figure S5), but the plasticizing effect was not jeopardized.

In the same vein, the binary mixtures of DEHF and DEHT plasticizers reported here, compared with the single use of DEHF as plasticizer [19], clearly have enhanced flexibility, since they had lower *E′* [19]. For example, PVC-DEHF (50 phr) presented an *E′* equal to 91 MPa at 23 °C [19]. In this respect, the DEHF/DEHT binary system seems to be not only a valuable approach in view of progressively replacing fossil-based plasticizers, but also due to their thermomechanical properties.

Table 2 summarizes the Young’s modulus, tensile strength, and elongation at break of all plasticized PVC blends. The most interesting result observed was the fact that the elongation at break of DEHF/DEHT based formulations increased from 247% in PVC-DEHT (PVC1) to 330% in PVC-DEHF/DEHT (PVC3), and tensile strength increased from 13.19 MPa (PVC1) to 17.46 MPa (PVC3), respectively, indicating an enhancement of the flexibility of PVC blends.

However, in the case of the highest amounts of DEHF used, in particular in the case of PVC5, the Young’s modulus and the tensile strength increased and the elongation at break decreased accordingly (Table 2), most probably associated with the highest incorporation of stiff furan moieties, as previously mentioned. This latter evidence was perfectly aligned with the DMTA results, showing that a DEHF content higher than 10 phr (PVC4-5) can change the final mechanical properties.

As expected, comparing DEHF/DEHT plasticized PVC (PVC2-5) with the non-plasticized one [15], as well as with DEHT-based PVC film (PVC1 formulation), an improvement on the flexibility was achieved, but at the expense of the Young’s modulus decrease (see Figure S6 of Supplementary Materials). In addition, enhanced flexibility was also achieved for the whole range of DEHF/DEHT ratios compared with PVC-DEHF with similar plasticizer content, displaying a maximum increase on the elongation at break of around 75% [19].

### 3.3. Thermal Properties of the PVCs and the Plasticizers Thereof

DEHF and DEHT plasticizers, as well as all the related PVC formulations were characterized in terms of their thermogravimetric behavior through TGA analysis, and the main results are summarized in Table 3 and Figure S7 of Supplementary Material.

TGA thermograms of DEHF/DEHT-based PVC films (PVC1 to PVC5) exhibited two major decomposition steps, (Figure S7), in agreement with other plasticized PVC systems [12,47,48]. The first step was within 200–380 °C, showing the highest weight loss of the PVC films (around 72%), and was related to de-chlorination of PVC and formation of polyenes, as well as to the thermal decomposition of the plasticizers. The second degradation step was found between 420 and 530 °C, with an observed lower weight loss (around 13%), and it occurred mainly due to the evolution of toluene and methylated aromatics coming from the decomposition of the polyenes [49].

For the whole range of DEHF/DEHT ratios used in the blends, an expected increase of the *T_d,max1_* compared with pure PVC (286 °C) was observed [47,49]. This was directly related to the incorporation of high-thermal behavior plasticizers in the blends. Indeed, the maximum degradation temperatures of DEHF and DEHT plasticizers were ca. 349 and 361 °C, respectively. In terms of *T_d,max2_*, no relevant variation was observed, except for a general decrease of this parameter in the case of the blends compared with the pure PVC counterpart.

Moreover, a decrease on both *T_d,5%_* and *T_d,10%_* of the plasticized PVC films compared with the non-plasticized PVC was noted, mostly due to the lower evaporation/degradation temperature of the DEHF plasticizer. Despite these results, the ensuing DEHF/DEHT-based PVC films, based on their *T_d,5%_* values, had higher thermal stability than those prepared from a single plasticizer, either DEHF [19] or DEHT (PVC1), in agreement with the occurrence of strong interactions between the mixture of the plasticizers and PVC matrix, favoring the blend stability. In summary, based on the *T_d,5%_* results, the new DEHF-PVC plasticized blends are thermally stable up to 243 °C, indicating their maximum working temperature.

### 3.4. Migration Resistance Tests of the PVCs

**Figure 5:**
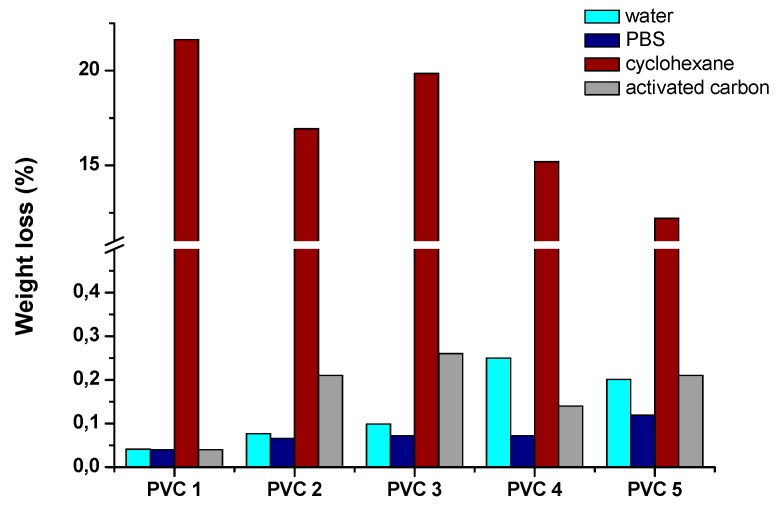
PVC blends weight loss percentage results determined from the leaching and volatile resistance tests. Error deviations were less than or equal to 0.01% (n = 3).

These new PVC blends were evaluated in terms of plasticizers migration stability, simulating potential DEHF/DEHT migration under daily situations, such as, for example, exudation from food/beverages packages and medical blood bags. In practice, the weight loss percentages by leaching of plasticizers from PVC specimens to distilled water, sodium phosphate buffer (pH ~7) and to cyclohexane were determined (leaching resistance) [50]. Additionally, the PVC blends were buried in active carbon and the PVC weight loss assessed (volatile resistance), simulating, for example, the case of plasticizer migration from baby nipples to solid medium. The main results of weight loss are summarized in Figure 5 and Table S3.

The weight loss percentages of all DEHF/DEHT plasticized PVC blends in both distilled water and PBS solution were very low, not exceeding ca. 0.3% and 0.2%, respectively, due to the hydrophobic nature of the plasticizers [51]. Kastner et al. [51] published slightly higher results for DEHT leaching in water after 1 and 3 weeks (0.79% and 1.91%, respectively), probably due to the longer period of the leaching experiment.

While both leaching in water and PBS increased with DEHF content in the binary systems, most probably due to a higher affinity of DEHF towards water than DEHT. In general terms, one can, however, mention that these findings indicated that the DEHF/DEHT mixture of plasticizers were quite compatible with the PVC matrix, in accordance with the FTIR results. Importantly, these low leaching values clearly showed that they were not easily extracted.

PVC-DEHT film (PVC1) had the lowest weight loss values in distilled water, followed very closely by all the other plasticized systems studied: PVC2 < PVC5 < PVC3 < PVC4. In general, all weight losses observed for PVC-DEHF/DEHT and PCV-DEHT samples were of the same magnitude as the previously published results, or even lower [11,12,13,44,45]. For example, migration tests in distilled water conducted in the same conditions as those carried out in the present study reported a weight loss of around 0.22% to PVC/DEHP, and of ca. 0.20% to a PVC/cardanol-based plasticizer blend [44].

An overall trend of the cyclohexane migration tests was that the weight loss results were much higher than those obtained in aqueous media (water or PBS), which is easily understood considering the essentially hydrophobic nature of both plasticizers and cyclohexane. The PVC-DEHT (PVC1) weight loss was higher than those obtained for the DEHF/DEHT-based films (PVC2-5), most probably due to a higher solubility of DEHT in cyclohexane. Similar tests using single DEHF also showed inferior weight losses of up to 9% [19]. This is very interesting as it enables the consideration of a new wide range of applications, especially for materials to be used in contact with foods with high fat content, such as, blood, and foods, among others.

Volatile resistance results (Figure 5 and Table S3) were quite similar to leaching resistance ones, displaying a maximum weight loss percentage of ca. 0.3% for PVC3 and the minimum weight loss percentage was observed for PVC1 (composed only of DEHT). A general trend of both leaching and volatile tests was that up to a DEHF/DEHT ratio equal to 10/45 phr/phr (PVC3), the weight loss percentages increased, but decreased thereafter for higher values of DEHF/DEHT ratios. This behavior was also in agreement with the thermal and mechanical results reported before. Moreover, in the case of PVC films plasticized with just DEHF [19], similar results were obtained (DEHF content > 30 phr). This trend could indicate, on the one hand, that for DEHF/DEHT ratios lower than 10/45 (PVC2 and 3), DEHT plasticizer dominated the interactions with the PVC matrix, whereas DEHF molecules acted as a secondary plasticizer. On the other hand, for the higher DEHF/DEHT ratios (higher than 10/45), DEHF plasticizer played a major role instead, and one could conjecture that it was also involved in relevant PVC-plasticizer interactions, hindering the migration process.

Importantly, the migration resistance tests of the newly prepared DEHF/DEHT binary mixtures showed weight loss percentage values quite below those of the DEHT benchmark (PVC1), which gives positive signs regarding their behavior under practical situations.

### 3.5. Cytotoxicity Assays of DEHF and DEHT

**Figure 6:**
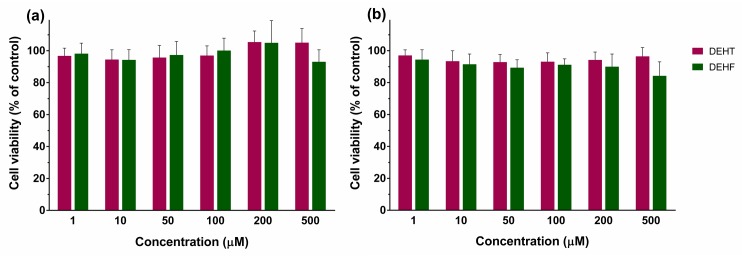
Cell viability in 3T3-L1 cell line after (**a**) 48 h and (**b**) 72 h of incubation in the presence of DEHF or DEHT plasticizers. Values represent the mean ± standard deviation (n = 9).

Toxicity is one of the majors health concerns associated with plasticizers routinely used in common “plastics” [52]. Actually, in this context, legislators have banned DEHP from toys and childcare articles [7]. The present study did not neglect this important issue and the cytotoxicity profile of DEHF, using the Alamar blue assay in 3T3-L1 cell line, was evaluated for the first time. For comparison reasons, cell viability in the presence of commercially used DEHT was also evaluated under the same conditions. The main results are displayed in Figure 6.

3T3-L1 mouse cells were incubated for 48 and 72 h in the presence of different amounts of DEHF and DEHT (ranging from 1 up to 500 μM, a typically high concentration) [53]. According to the Alamar blue assay results, plasticizers did not exhibit significant toxicity in 3T3-L1 cells, up to a concentration of 500 μM and for a period of 48 and 72 h. More than 90% cell viability was obtained with both DEHF, and as expected, with DEHT, after 72 h of incubation. This is a first preliminary indication of the biocompatibility of DEHF, despite the fact that further studies addressing the cell viability of the plasticizers’ metabolites are important to consider in the future [54]. Nevertheless, materials that promote cell viability higher than 80% are considered as biocompatible [55].

## 4. Conclusions

In summary, an effective strategy to prepare plasticized PVC blends with higher “green content”, less toxic profile, and interesting compatibility and properties, based on the combination of DEHF and DEHT compounds, is reported. The ensuing PVC/DEHF-DEHT materials were shown to have enhanced compatibility between PVC matrix-mixture of plasticizers, compared to the single use of DEHF [19], confirmed by FTIR spectroscopy. In this regard, all PVC blends spectra display a red-shift of the C=O stretching band from 1718 cm^−1^ to lower wavenumbers, in accordance with the occurrence of dipole-dipole interactions [36], and an effective plasticization process. Accordingly, these new PVC blends were shown to have a reduced *T_g_*, from around 97 to 20 °C, together with an enhanced elongation at break (not lower than 330%), but at the expense of some reduction of its stiffness with Young’s modulus of approximately 8 MPa. Importantly, migration tests showed promising results in terms of application prospects. For example, migration tests of PVC/DEHF-DEHT blends in cyclohexane, show improved results compared to PVC-DEHT, most probably due to a higher solubility of DEHT in cyclohexane. This is a very interesting fact enabling the consideration of a new wide range of applications, especially for materials to be used in contact with foods with high fat content, such as, blood and foods, among others. Cytotoxicity assays of DEHF (as well as DEHT), seldom ignored, but of upmost importance for assessing the plasticizer benign profile (or not) was performed in this study for the first time. Cell viability assays of DEHF (as well as DEHT), up to 500 μM, using 3T3-L1 cell line, for a maximum period of 72 h, 90% cell viability was observed. Biodegradation of DEHF using common soil organisms to simulate the end-life disposal of the plasticizer is under investigation. In conclusion, this study highlights the enormous interest in designing novel FDCA-based plasticizer systems and future directions also call for the development of 2,5-furandicarboxylate esters from linear alkyl alcohols [56], such as *n*-hexanol due to its more rapid biodegradation kinetics combined with similar plasticizer effectiveness when compared to 2-ethylhexanol [57].

## Figures and Tables

**Table 1:** Compositions of the different formulations of PVC-DEHF/DEHT prepared.

Components	Formulations					
	pure PVC	PVC1	PVC2	PVC3	PVC4	PVC5
	amount/g					
**PVC resin-K70**	40.0	40.0	40.0	40.0	40.0	40.0
	(100 phr) ^a^	(100 phr)	(100 phr)	(100 phr)	(100 phr)	(100 phr)
**DEHT**	-	22.0	20.0	18.0	16.0	14.0
		(55 phr)	(50 phr)	(45 phr)	(40 phr)	(35 phr)
**DEHF**	-	0.0	2.0	4.0	6.0	8.0
		(0 phr)	(5 phr)	(10 phr)	(15 phr)	(20 phr)
**Stearic acid**	-	0.12	0.12	0.12	0.12	0.12
		(0.3 phr)	(0.3 phr)	(0.3 phr)	(0.3 phr)	(0.3 phr)
**Zinc stearate**	-	0.40	0.40	0.40	0.40	0.40
		(1.0 phr)	(1.0 phr)	(1.0 phr)	(1.0 phr)	(1.0 phr)

^a^ phr—per hundred resin.

**Table 2:** Main results of Young’s modulus, elongation at break, and tensile strength determined at 25 °C.

Formulations	Young’s Modulus/MPa	Elongation at Break (%)	Tensile Strength/MPa
**Pure PVC**	153.87 ± 6.3 ^a^	180.37 ± 5.16 ^a^	30.33 ± 0.28 ^a^
**PVC1**	8.96 ± 0.26	246.64 ± 9.38	13.19 ± 0.55
**PVC2**	8.20 ± 0.14	316.26 ± 9.65	16.37 ± 0.51
**PVC3**	7.58 ± 0.29	330.34 ± 11.66	17.46 ± 0.54
**PVC4**	7.72 ± 0.45	238.63 ± 4.26	13.35 ± 0.24
**PVC5**	8.69 ± 0.31	225.76 ± 7.49	14.57 ± 0.37

^a^ Reference [47].

**Table 3:** Decomposition under N_2_ atmosphere at 5%, 10%, and 50% weight loss (*T_d,5%_*, *T_d,10%_* and *T_d,50%_*, respectively) and maximum decomposition (*T_d,max_*) temperatures of plasticized PVC films, and their pure components counterparts.

Formulations	*T_d, 5%_* (°C)	*T_d, 10%_* (°C)	*T_d, 50%_* (°C)	*T_d, max1_* (°C)	*T_d, max2_* (°C)	Weight Loss (%)		Residue (%)
						1st Step	2nd Step	
						200–380 °C	420–530 °C	
**DEHF**	175.9	237.8	318.8	349.8	-	-	-	0.01
**DEHT**	279.0	297.3	343.1	360.5	-	-	-	0.07
**Pure PVC**	268.7	274.6	303.2	286.4	461.7	58.2	23.7	3.46
**PVC1**	241.9	262.0	308.2	305.0	455.7	71.8	13.5	8.85
**PVC2**	243.0	263.3	308.5	298.7	460.3	72.1	12.8	9.93
**PVC3**	250.0	262.7	306.1	297.5	459.4	71.7	12.6	13.22
**PVC4**	245.1	264.0	308.0	295.1	459.2	72.6	12.6	10.44
**PVC5**	247.3	263.2	304.6	292.3	458.3	72.2	12.1	12.75

## Supplementary Materials

The following are available online at https://www.mdpi.com/1996-1944/12/14/2336/s1, Figure S1: ATR FTIR spectra of DEHF plasticizer (left) and of DEHF/DEHF (20/35 phr/phr) mixture of plasticizers (right); Figure S2: ^1^H NMR spectrum of DEHF (CDCl_3_); Figure S3: ^13^C NMR spectrum of DEHF (CDCl_3_); Figure S4: Main results from GC-MS analysis (a) GC chromatogram and (b) MS spectra of DEHF; Figure S5: *E´´* traces of all PVC films; Figure S6: Mechanical properties of all PVC-DEHF/DEHT blends: (a) Young’s modulus, (b) elongation at break and (c) tensile strength; Figure S7: TGA (a) and DTG (b) thermograms of all PVC-DEHF/DEHT blends, related DEHF and DEHT plasticizers and pure PVC.; Table S1: Chemical characteristics of the different plasticizers; Table S2: Glass transition (*T_g_*) and storage modulus (*E′*) at −10 °C and 25 °C of all PVC-DEHF/DEHT blends; Table S3: PVC blends weight loss percentage results determined from the chemical and volatile resistance tests.
