# Supplementary material for: Replacing Di(2-ethylhexyl) Terephthalate by Di(2-ethylhexyl) 2,5-Furandicarboxylate for PVC Plasticization: Synthesis, Materials Preparation and Characterization
Source: Materials (Basel). 2019 Jul 23;12(14):2336. doi: 10.3390/ma12142336 (PMC6678310; doi:10.3390/ma12142336)
Supplement: Supplementary file 1 [file materials-12-02336-s001.pdf]

materials

Supplementary Materials: Replacing Di(2-ethylhexyl) Terephthalate by Di(2-ethylhexyl) 2,5-Furandicarboxylate for PVC Plasticization: Synthesis, Materials Preparation and Characterization

Marina Matos [1], Rosemeyre A. Cordeiro [2], Henrique Faneca [2], Jorge F.J. Coelho [3], Armando J.D. Silvestre [1] and Andreia F. Sousa [1,*]

[1] CICECO-Aveiro Institute of Materials and Department of Chemistry, University of Aveiro, 3810-193 Aveiro, Portugal
[2] Center for Neuroscience and Cell Biology, University of Coimbra, 3004-504 Coimbra, Portugal
[3] CEMMPRE, Department of Chemical Engineering, University of Coimbra, 3030-790 Coimbra, Portugal
* Correspondence: andreiafs@ua.pt; Tel.: +351-234-401-470

Received: 31 May 2019; Accepted: 19 July 2019; Published: 23 July 2019

1. Structural Characterization

Table S1. Chemical characteristics of the different plasticizers.

Plasticizers	Molecular Formula	Molecular Weight	Viscosity [a] (cP; at 25 °C)	Density (g cm^{-3})
**DEHF**	$C_{22}H_{36}O_5$	380.53	55	0.98
**DEHT**	$C_{24}H_{38}O_4$	390.56	63	0.98

[a] The relative uncertainty of the dynamic viscosity is ±0.35%.

1.1. ATR FTIR Results

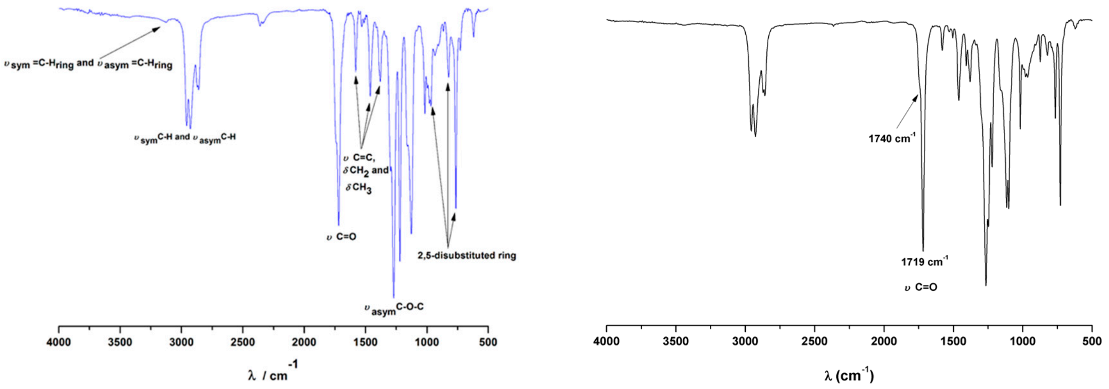

Figure S1. ATR FTIR spectra of DEHF plasticizer (left) and of DEHF/DEHF (20/35 phr/phr) mixture of plasticizers (right).

materials

1.2. NMR Results

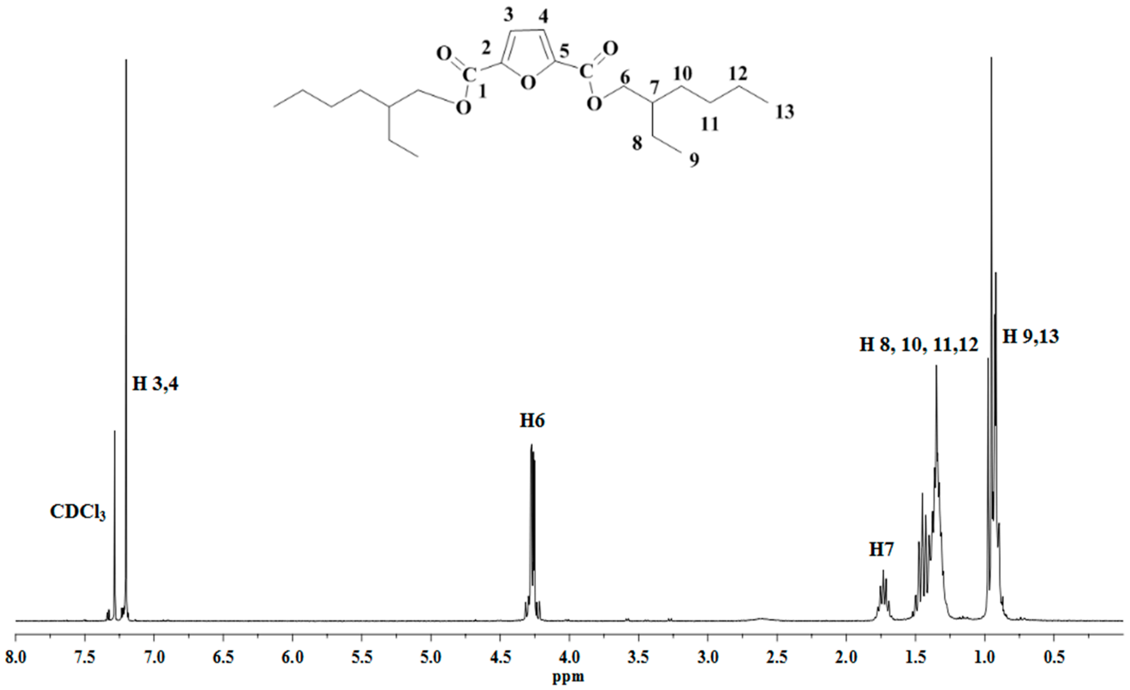

Figure S2. ^{1}H NMR spectrum of DEHF ($CDCl_3$).

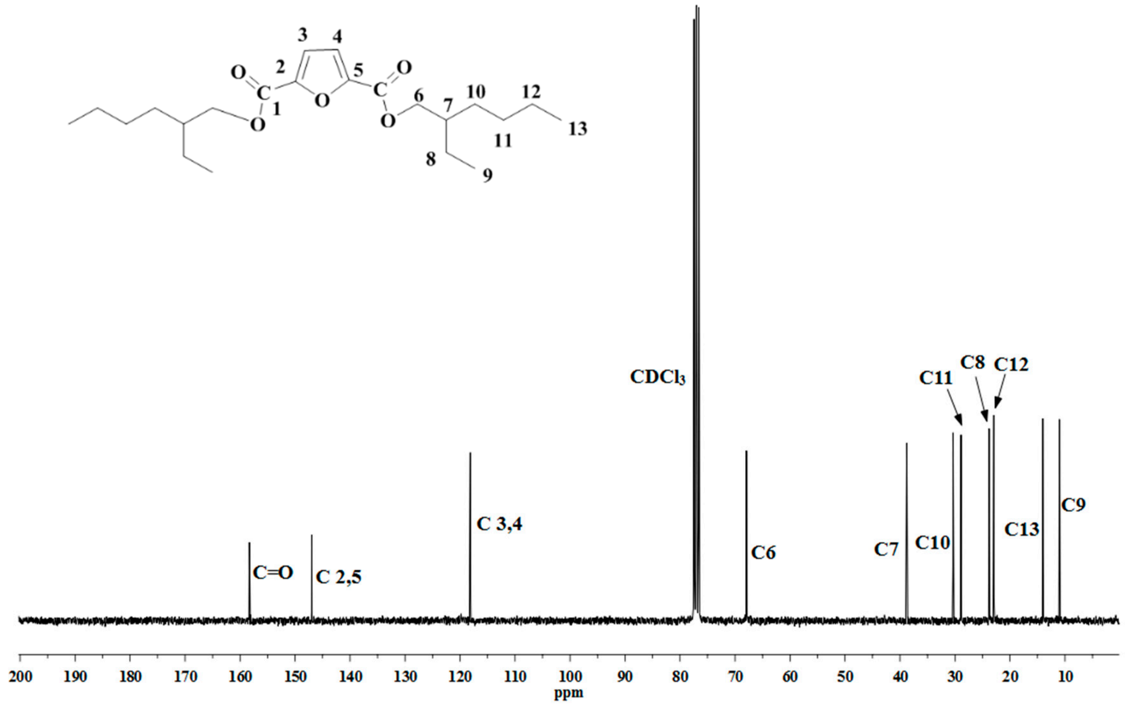

Figure S3. ^{13}C NMR spectrum of DEHF ($CDCl_3$).

materials

1.3. GC-MS Results

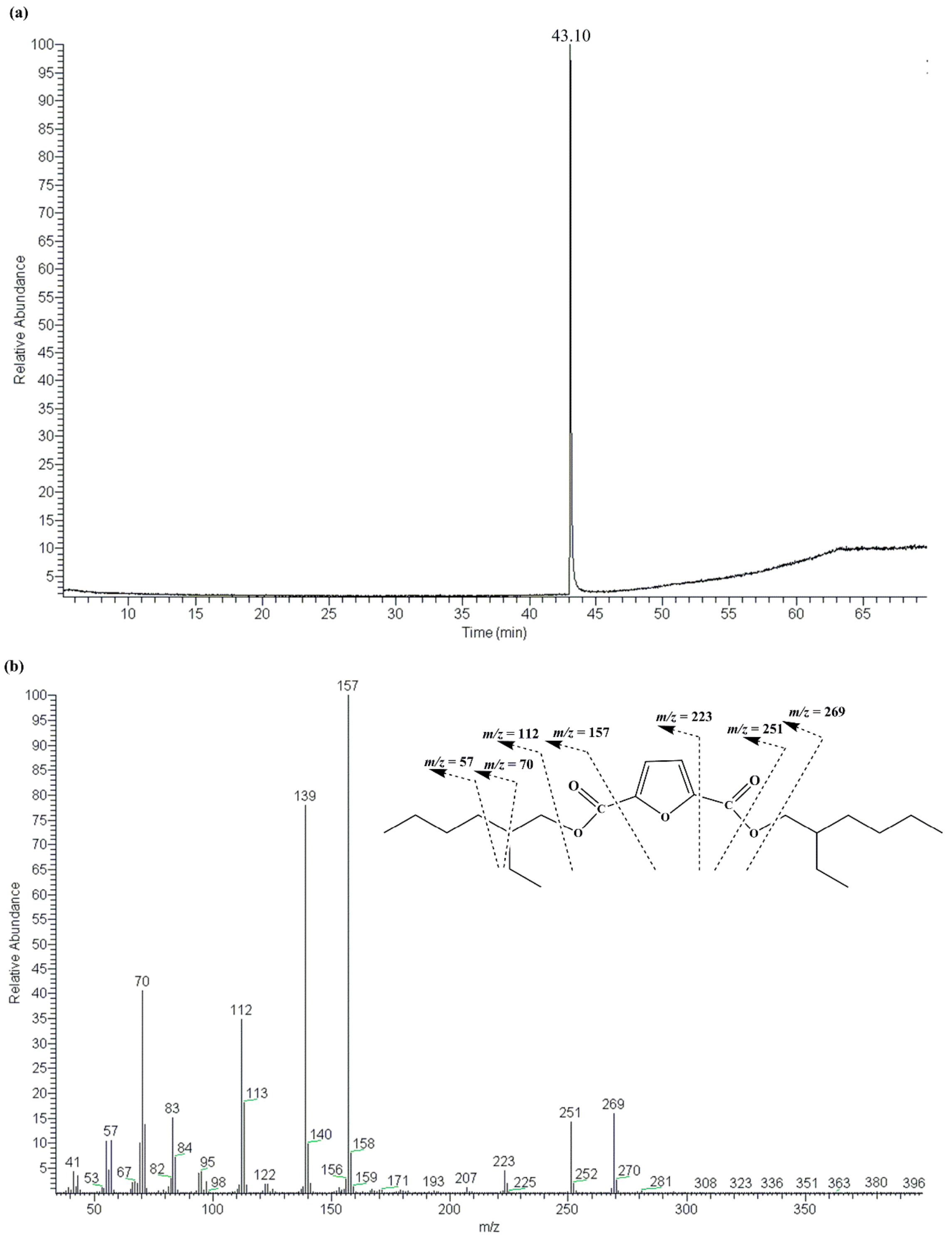

Figure S4. Main results from GC-MS analysis (**a**) GC chromatogram and (**b**) MS spectra of DEHF.

materials MDPI

2. Mechanical Properties

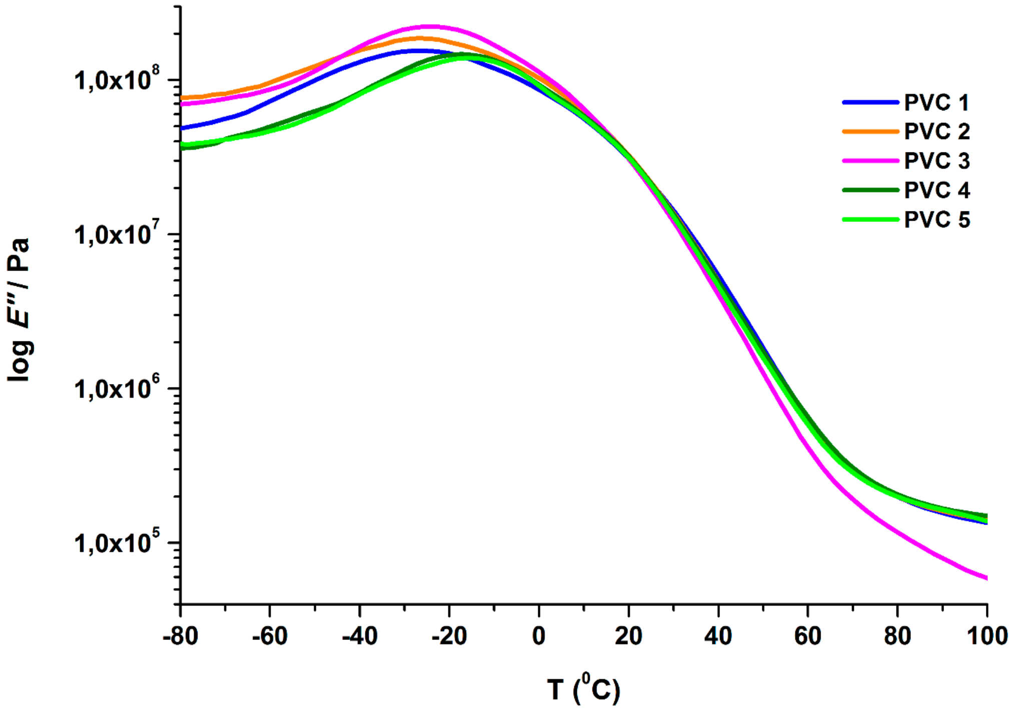

Figure S5. *E´´* traces of all PVC films.

Table S2. Glass transition (T_g) and storage modulus (E') at −10 °C and 25 °C of all PVC-DEHF/DEHT blends.

Formulations	T_g (°C)	E' (MPa)		
		at $T_{-10°C}$	at T_g	at $T_{25°C}$
**Pure PVC**	97.4[a]	-	-	-
**PVC 1**	23.5	551.8	50.6	42.9
**PVC 2**	22.1	658.2	56.9	42.2
**PVC 3**	20.4	694.7	58.5	38.2
**PVC 4**	21.1	617.3	55.2	39.4
**PVC 5**	21.5	649.7	56.1	40.2

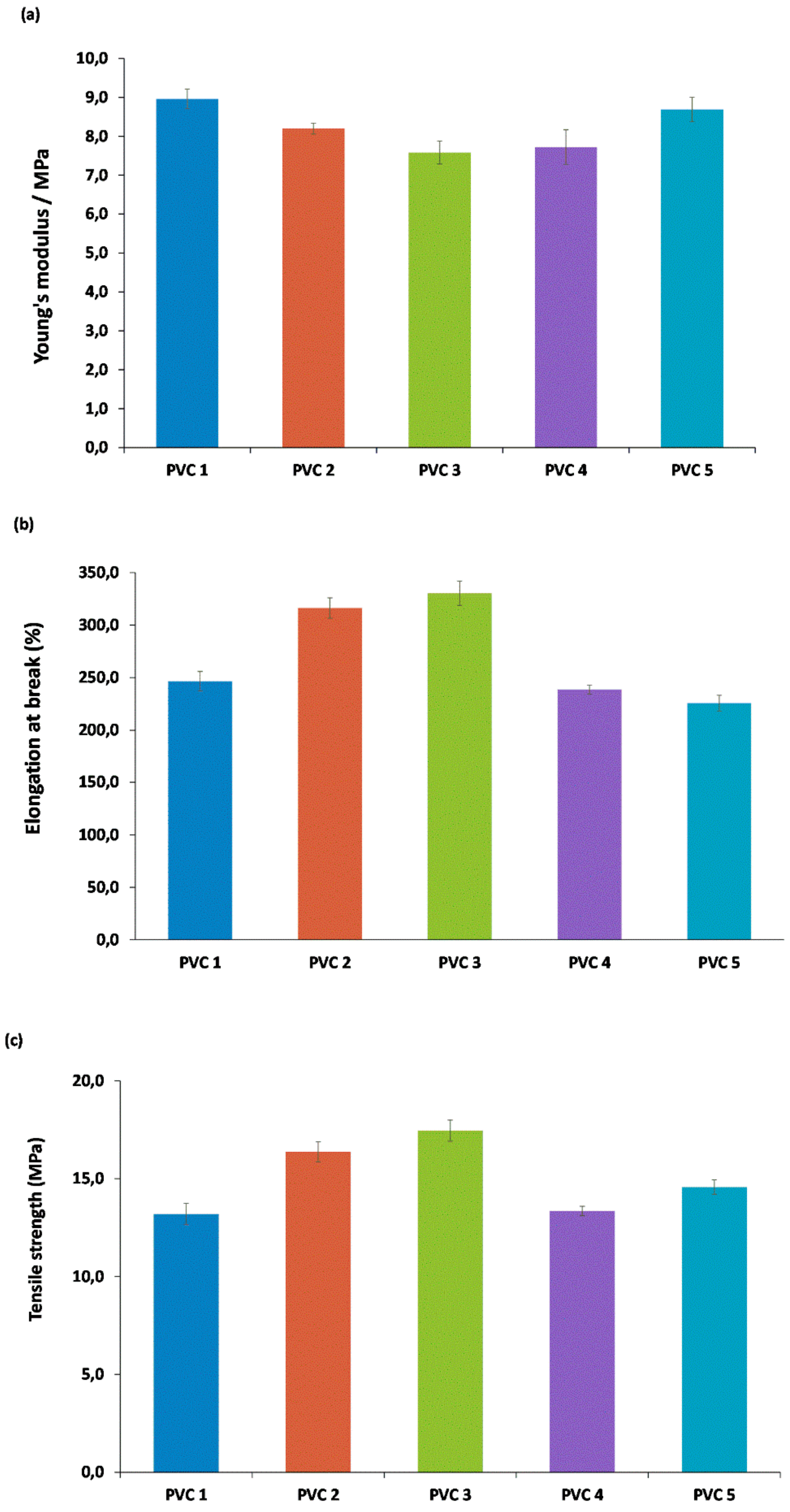

Figure S6. Mechanical properties of all PVC-DEHF/DEHT blends: (**a**) Young's modulus, (**b**) elongation at break and (**c**) tensile strength.

materials

3. Thermal Properties

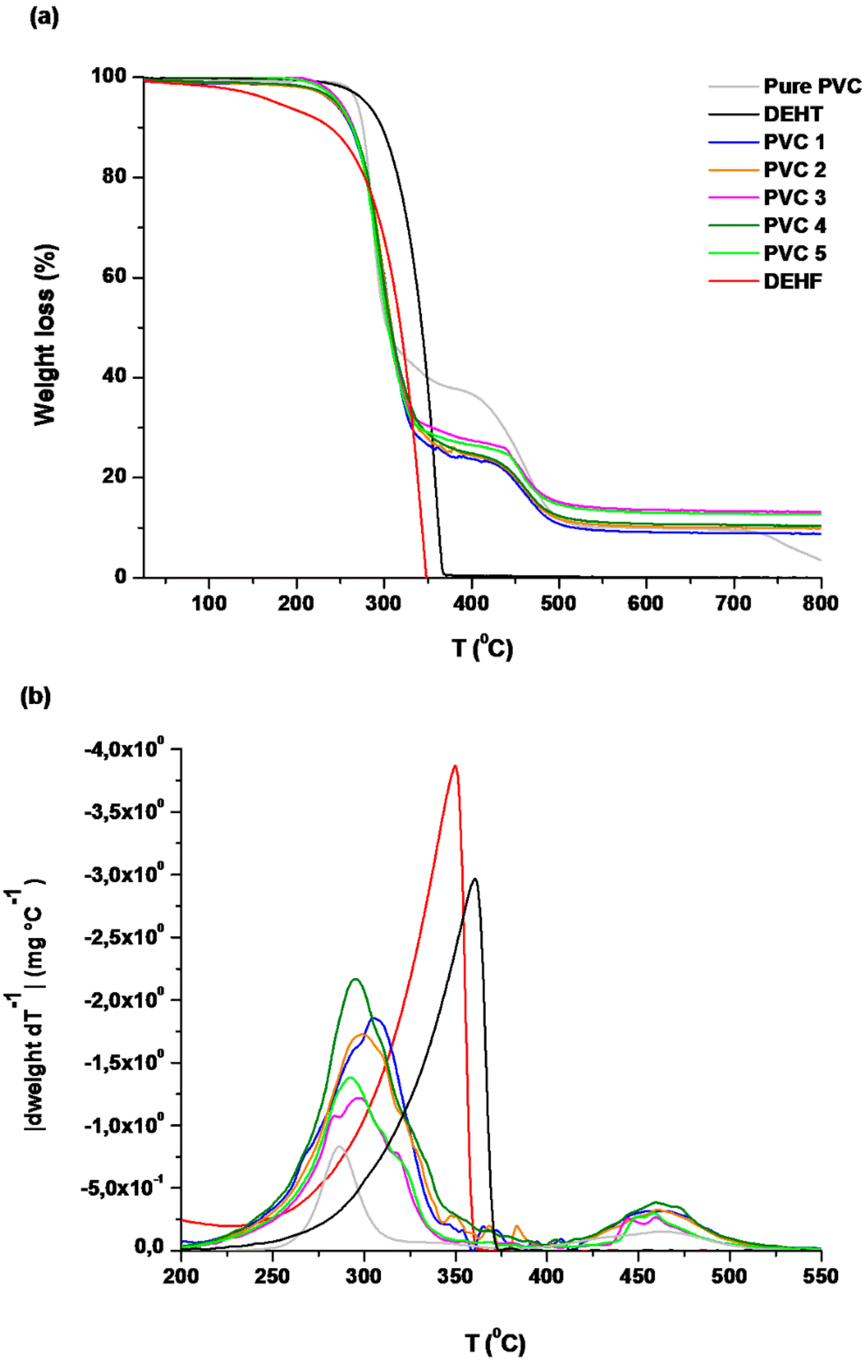

Figure S7. TGA (**a**) and DTG (**b**) thermograms of all PVC-DEHF/DEHT blends, related DEHF and DEHT plasticizers and pure PVC.

materials

4. Migration Resistance Tests

Table S3. PVC blends weight loss percentage results determined from the chemical and volatile resistance tests.

Chemicals	Formulations				
	PVC1	PVC2	PVC3	PVC4	PVC5
	weight loss (%) [a]				
**water**	0.04	0.08	0.26	0.27	0.09
**PBS**	0.04	0.07	0.17	0.13	0.13
**cyclohexane**	21.40	16.93	19.86	15.19	12.20
**activated carbon**	0.04	0.21	0.26	0.14	0.21

[a] Error deviations were less than or equal to 0.01%.
